# Clinical characteristics of the host DNA-removed metagenomic next-generation sequencing technology for detecting SARS-CoV-2, revealing host local immune signaling and assisting genomic epidemiology

**DOI:** 10.3389/fimmu.2022.1016440

**Published:** 2022-11-15

**Authors:** Sun Zhaoyang, Song Guowei, Pan Jing, Zhou Yundong, Lu Xinhua, Wei Muyun, Ma Xiaowei, Li Lixin, Chen Xiaoying

**Affiliations:** ^1^ Department of Laboratory Medicine, Ren Ji Hospital, Shanghai Jiao Tong University School of Medicine, Shanghai, China; ^2^ Department of Laboratory Medicine, Shijiazhuang People’s Hospital, Shijiazhuang, China; ^3^ Shanghai Medical Innovation Fusion Biomedical Research Center, Shanghai, China

**Keywords:** mNGS, RT-PCR, SARS-CoV-2, the removal of host, traceability

## Abstract

**Background:**

Metagenomic next-generation sequencing (mNGS) technology has been central in detecting infectious diseases and helping to simultaneously reveal the complex interplay between invaders and their hosts immune response characteristics. However, it needs to be rigorously assessed for clinical utility. The present study is the first to evaluate the clinical characteristics of the host DNA-removed mNGS technology for detecting SARS-CoV-2, revealing host local immune signaling and assisting genomic epidemiology.

**Methods:**

46 swab specimens collected from COVID-19 patients were assayed by two approved commercial RT-qPCR kits and mNGS. The evolutionary tree of SARS-CoV-2 was plotted using FigTree directly from one sample. The workflow of removing the host and retaining the host was compared to investigate the influence of host DNA removal on the performances of mNGS. Functional enrichment analysis of DEGs and xCell score were used to explore the characteristics of host local immune signaling.

**Results:**

The detection rate of mNGS achieved 92.9% (26/28) for 28 samples with a Ct value ≤ 35 and 81.1% (30/37) for all 46 samples. The genome coverage of SARS-CoV-2 could reach up to 98.9% when the Ct value is about 20 in swab samples. Removing the host could enhance the sensitivity of mNGS for detecting SARS-CoV-2 from the swab sample but does not affect the species abundance of microbes RNA. Improving the sequencing depth did not show a positive effect on improving the detection sensitivity of SARS-CoV-2. Cell type enrichment scores found multiple immune cell types were differentially expressed between patients with high and low viral load.

**Conclusions:**

The host DNA-removed mNGS has great potential utility and superior performance on comprehensive identification of SARS-CoV-2 and rapid traceability, revealing the microbiome’s transcriptional profiles and host immune responses.

## Introduction

Infectious diseases have been, and still are, a leading cause of human morbidity and mortality worldwide and are also a tremendous challenge for the biomedical sciences. Accurate and rapid diagnosis of infectious diseases will be of great significance for reducing the medical therapies burden on patients, straining the increasingly drug-resistant organisms and standardizing antibiotic stewardship (1, 2). However, clinical diagnosis of infectious diseases is often characterized as complex and difficult for the following critical reasons: (a) Many abnormal indicators caused by suspected infection may be part of symptoms of complicated underlying disease; (b) The human pathogens are so rich and diverse that it is difficult to explicitly definite the species of the suspected pathogen.

Traditional diagnostic techniques in the microbiology laboratory include culture techniques, detection of pathogen-​specific antibodies (serology) or antigens, and molecular identification of microbial nucleic acids (DNA or RNA), most commonly *via* PCR (3–5). However, these techniques detect only one or a small number of pathogens in a given reaction (6). Comprehensive screening of all species of pathogens is extremely important for the precision diagnosis and therapy of infectious disease and is also part of precision medicine, which requires precision at all levels. Considering its paramount clinical importance, improving microbiological diagnosis needs more reliable detection technologies. In recent years, untargeted metagenomic next-generation sequencing (mNGS) has emerged as a promising technique because of its special strengths and abilities for comprehensively detecting all pathogens in samples (7–9). Compared with most traditional diagnostic techniques that only target a limited number of pathogens using specific primers or probes or specific antigens, metagenomic approaches characterize all DNA or RNA present in a sample, enabling analysis of the entire microbiome as well as the human host genome or transcriptome in patient samples (3).

However, the clinical application of mNGS is still in its early stages and is not yet routinely established in the clinical environment. There are also no uniform criteria for pathogen identification by mNGS because of its extremely high level of complexity in the entire detection process (10). The draft guidance issued by Food and Drug Administration (FDA) points out exactly that the clinical performance characteristics of NGS technology for microbial identification lie in its limit of detection (LOD), inclusivity, interfering substances, repeatability, cross-contamination and stability (11). These indicators used to evaluate the detection performance of mNGS require more comprehensive and in‐depth studies.

Emerging pieces of evidence demonstrated that mNGS could yield a higher sensitivity for pathogen identification than conventional culture-based techniques and has sensitivity similar to specific PCR assays (12–14). Interestingly, unlike current traditional diagnostic techniques, the sensitivity of mNGS for pathogens detection is affected by a series of variables: efficiency of nucleic acid extraction (bias toward some species), pathogen genome size (at the same organism load, more reads are generated from longer genomes), the robustness of library preparation, the total number of sequences reads generated from a given specimen (more reads ≈higher sensitivity), specimen composition and background reads, bioinformatics pipeline used for analysis (availability of appropriate reference sequences in databases), sequence similarity with related organisms (confident differentiation of close relatives requires greater sequencing depth than the identification of unique sequences), the accuracy of classification algorithms, and required confidence for pathogen identification (15).

For the reasons above, our present study aims to evaluate the sensitivity of DNA-removed mNGS by detecting 46 swab sample from patients with COVID-19 infection and comparing the mNGS and two approved quantitative real-time PCR (qRT-PCR). In addition, our results will also provide further insight into understanding the superior performance of DNA-removed mNGS in the comprehensive identification of the pathogen and simultaneously reveal the transcriptional profiles of the microbiome and host responses.

## Methods

### Swab specimen collection from the hospital in patients with COVID-19 infection

46 swab specimens were collected from inpatients diagnosed with COVID-19 infection from Shijiazhuang People’s Hospital. All patients were treated in isolation between January 2021 and March 2021. This study has been approved by Shijiazhuang People’s Hospital Ethics Committee. The Ethics Approval Number: [2020]-046.

### Nucleic acid extraction of swab specimen

The nucleic acid was extracted from a 200µl swab sample using an automatic nucleic acid extraction instrument (Smart Lab Assist) and its supporting reagents (Taiwan Advanced Nanotech, Taiwan, China) according to the manufacturer’s protocol. Isolated nucleic acid was eluted in a 50µl elution buffer. Then, 33µl nucleic acid from each swab sample was used to performed the mNGS assay, and 5µl was performed for qRT-PCR detection by using two commercial RT–PCR kits-DAAN and BioGerm, which have been both approved by the China National Medical Products Administration (NMPA).

### SARS-CoV-2 detection by two different clinical RT-PCR kits

The primers of two RT–PCR kits were both targeted to the regions of the SARS-Cov-2 N gene and ORF1ab gene. According to the judgment criteria of two RT–PCR kits, cycle threshold (Ct) values below 40 were regarded as positive and above 40 as negative.

### The schematic flow of mNGS detection

The detection process of untargeted host-removed mNGS is as follows. (a) After extracting the nucleic acid from the swab specimen, 33µl nucleic acid of each sample was mixed with 3uL DNA enzyme and DNA enzyme buffer to digest DNA and enrich RNA. (b) Reverse transcription and cDNA synthesis. (c) cDNA library preparation using the PMseq RNA infectious pathogens high throughput detection kit (probe anchored polymer sequencing method) (Green Pine Capital Partners Co. LTD, Wuhan, China). The qualification of the cDNA libraries concentration was quantified using the Qubit4.0. (d) The DNB (DNA nano ball) was prepared after the qualification of cDNA libraries and then loaded into the sequencing chip. (e) Sequencing was performed on the MGSEQ-2000 platform (MGI Tech Co., Ltd. Shenzhen, China, https://en.mgi-tech.com/about/). The sequence was generated with a single-end, 50 bp size reading (SE50). We defined samples with positive SARS-CoV-2 results when the specific reads of SARS-CoV-2 detected from samples was greater than or equal to 1. Specific reads of SARS-CoV-2 were those mapped exclusively to SARS-CoV-2 species, to discriminate those aligned to other species.

### Bioinformatic analysis

After the sequencing was completed, we first removed adaptor sequences from raw reads and discarded low-quality reads. Then, two different bioinformatics analysis workflows were performed to analyze the transcriptome sequence profile of human and the sequence information of microbial species in swab samples, respectively. For microbiota analysis workflow, the sequences aligned to the human reference genome were removed, followed by comparing the microbiota sequences with the reference genome sequences in the database to determine the microbial species information. The sequence of SARS-CoV-2 was extracted and used to assemble the viral genome, followed by aligning the full genome sequences with reference genomes derived from NCBI. Then the phylogenetic trees were constructed using the Maximum Parsimony method included in evolutionary tree analysis software MEGA based on the 50 optimum alignment genomes. For analysis workflow of human transcriptome sequence, we screened raw data to make clean data by removing contaminants, adaptors, low-quality reads using the Trimmomatic program (version.0.39) (https://github.com/timflutre/trimmomatic), which removed the leading and trailing low-quality bases below quality 3 or N bases, cut the sliding window which average quality per base drops below 15, and dropped reads below the 36 bases long. A quality control using FastQC was performed on the reads (https://www.bioinformatics.babraham.ac.uk/projects/fastqc/, v0.11.9). Then, sequences were aligned to the reference human genome version GRCh38 (Gencode, version 39) (15). Transcript abundance was computed using Salmon version 1.8.0 (16).

### Data analysis and statistics

Data analyses were performed using R statistical language (version 4.1.0) and Origin 2018 64Bit. Comparison of the test results between the host removed and the host retained workflow was tested using two-tailed paired t-test. GO enrichment is visualized using the GOplot R package. Differential gene expression and signature enrichment analysis were performed using a two-sided Wilcoxon rank sum test, and statistical significance was defined as *P* <0.05.

## Results

### Compared with two approved RT-qPCR kits, mNGS presented credible sensitivity for detecting SARS-CoV-2 from swab samples

**Figure 1:**
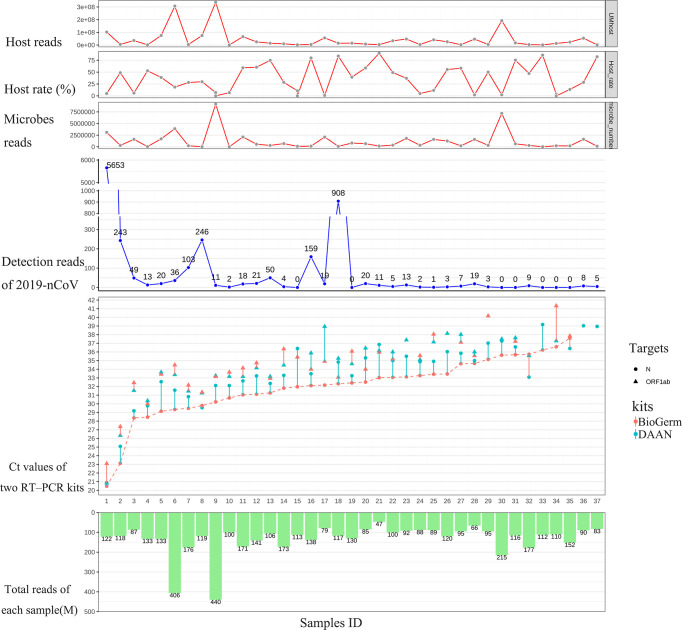
Results comparison between mNGS and two approved RT-qPCR methods. From left to right, the 37 samples were sorted according to the Ct value of N gene from DAAN (red curve). From top to bottom are the host reads, the percentage of host read in each sample, microbes read in each sample, detection read number of 2019-nCoV, Ct values of two RT-PCR kits and total reads (including the reads from host and microbes in swab sample) generated from each sample.

To strengthen the reliability of the results, we compared the detection rate of mNGS for detecting SARS-CoV-2 with two approved commercial RT-qPCR kits, both approved by NMPA and widely used for risk screening of COVID-19 in the majority of health care institutions across China. The detailed results of 46 swab samples derived from mNGS and two RT-qPCR detections are given in **Supplementary Table 1**. Among 46 swab samples from 46 hospitalized patients, the result of the 9 sample (Samples 38-46) was simultaneously confirmed to be negative by two different RT-PCR kits based on the judging criteria; the remaining 37 samples were confirmed to be positive. As shown in **Figure 1**, among 37 samples that showed positive RT-PCR results, 30 (30/37, 81.1%) samples were found the SARS-CoV-2 sequences (reads≥1) and were considered to be consistent with the results of RT-PCR assays; residual 7 (7/37, 18.9%) samples with positive RT-PCR results fail to detect the SARS-CoV-2 sequence. Notably, for RT-PCR positive samples with Ct value ≤ 35, the positive rates of mNGS was 92.9% (26/28).

### The relationship between the total reads and the genome coverage of SARS-CoV-2

**Figure 2:**
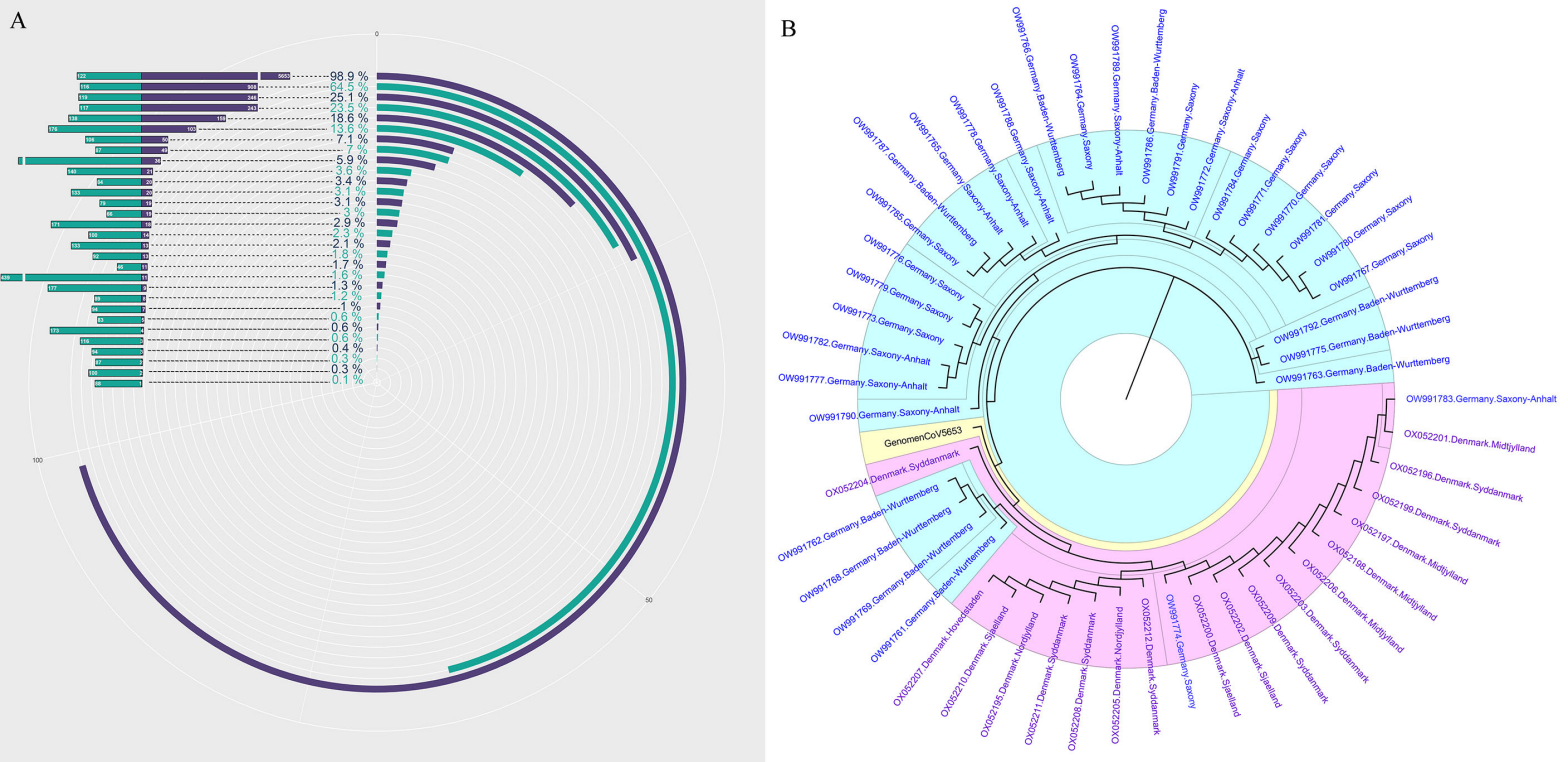
The genome of SARS-CoV-2 analysis based on mNGS. **(A)** The genome coverage of SARS-CoV-2 increases with the increasing reads of SARS-CoV-2. **(B)** The evolutionary tree analysis between our sample-GenomenCoV5653 and the 50 optimal genome alignment from NCBI.

Recent advances in next-generation sequencing (NGS) technologies have markedly increased the amount of data (=Total reads, 1M=10^7^reads) produced by a single sample in each test and significantly reduced the sequencing cost. However, almost no previous research has elucidated the clinical and research significance of increased total data amount. In this study, we first revealed the relationship between the amount of data (total reads) and the genome coverage of SARS-CoV-2. The 30 samples with positive mNGS results were ranked according to the detection reads of SARS-CoV-2. The result is consistent with the theory, which suggests that the genome coverage of SARS-CoV-2 increases with the increasing reads of SARS-CoV-2. However, it was unexpected that the genome coverage of SARS-CoV-2 could reach up to 98.9% when the reads number of SARS-CoV-2 was 5653 in the sample, corresponding to the Ct value of approximately 20 (**Figure 2A**).

Next, the sample with the highest coverage (98.9%) of SARS-CoV-2 was conducted genome assembly using MUSCLE (http://www.ebi.ac.uk/Tools/msa/muscle/). The assembled contigs sequence was aligned with the online NCBI alignment tools to obtain the 50 optimal genome alignment. Then, phylogenetic trees were constructed using the MEGA software to explore their evolutionary relationship in terms of geographical locations. The genomic epidemiology analysis showed that our sample-GenomenCoV5653 presents a close orthologous relationship with the genomes of the viruses from Germany (**Figure 2B**).

### Comparison results from the workflow of removing host and workflow of retaining host

**Figure 3:**
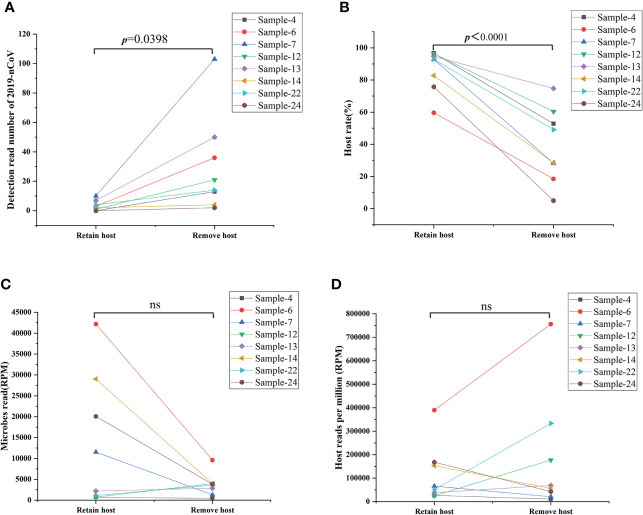
Comparison results from removing host and retaining host workflow. **(A)** Removing the host significantly improved the detection read number of 2019-nCoV (*p*<0.05). **(B)** Removing the host decreased significantly the host rate in the swab sample (*p*<0.05). **(C, D)** Removing the host on the detection reads of microbes reads and the host reads varies in different samples. RPM: Numbers of mapped reads per million; ns: no significance.

As an unbiased detection method, mNGS could efficiently detect all RNA in the sample without bias, including all RNA from microbes and hosts. Removing the host DNA could theoretically improve the sensitivity of this technique for the analysis of all RNA in the sample. However, little evidence exists to demonstrate whether such removal of host DNA will cause the loss of part of RNA form viruses, microbes, and hosts in the sample. Given this, our study represents the first investigation of the efficacy and influence of host DNA removal for analyzing all RNA in the sample. Eight samples were selected and simultaneously performed the remove host and retain host process. As shown in **Figure 3**, the procedure of removal host can significantly improve the detection read number of 2019-nCoV (**Figure 3A**) and decrease the host rate (**Figure 3B**) in all 8 samples when comparing the procedure of retaining the host (*p*<0.05). In contrast, the microbes’ reads (**Figure 3C**) and the host reads (**Figure 3D**) do not present the same changing trend after removing the host for an identical sample (ns: no significance). These results demonstrate that the removal of the host could enhance the sensitivity of mNGS for detecting SARS-CoV-2 from swab sample

### Removing the host does not affect the species’ abundance of microbes RNA

**Figure 4:**
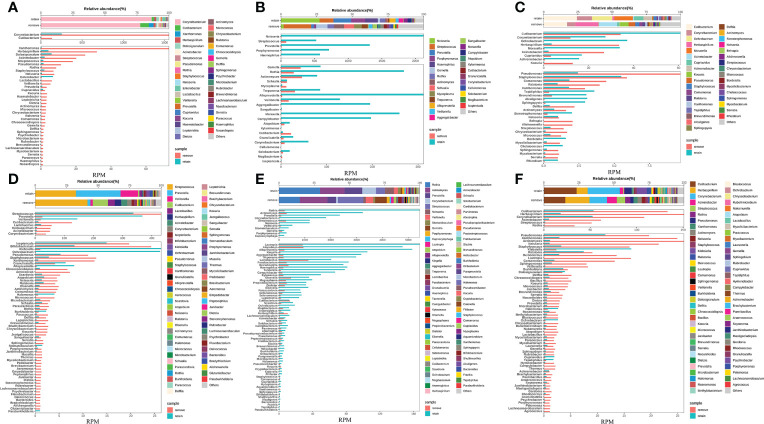
Removing host DNA detection workflow show identical species abundance of bacteria with the workflow of retaining host DNA and will not cause the loss of RNA from bacterial species. **(A-F)** Eight samples simultaneously performed the workflow of removing and retaining host DNA and compared the bacterial species composition between the two workflows.

To enhance the sensitivity of untargeted mNGS for detecting and analyzing the RNA from viruses and transcriptomic information from hosts and microbes, our study removed host DNA by introducing DNA enzymes before library preparation. We have demonstrated that the removal of host DNA positively affects the detection of virus RNA in the sample based on the result above. However, it is unclear that whether this method will cause the untargeted degradation of microbes’ RNA and influence the microbe species’ abundance based on the analysis of microbes’ RNA. Given this, our study first compared species abundance in removing host DNA and retaining host DNA at the RNA level in eight pairs of samples. Our results found that the species, proportion, and abundance of microbe in removing and retaining host DNA were almost identical (**Figures 4A–F**) (**Figures S2A, B**), demonstrating that the removal of the host does not affect the species abundance of microbes RNA.

### Improving the sequencing depth did not show a positive effect on improving the detection sensitivity of SARS-CoV-2

**Figure 5:**
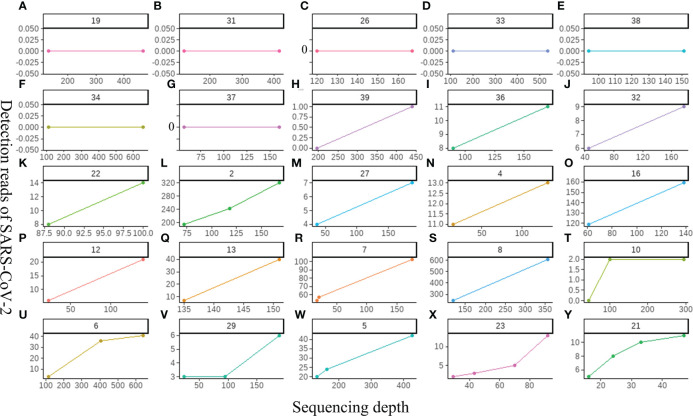
The relationship between the sequencing depth and the detection sensitivity of SARS-CoV-2 based on untargeted host-removed mNGS technology. **(A-Y)** 25 samples were assayed by mNGS using sequencing depth, respectively.

Our study first explored the relationship between the sequencing depth and the detection sensitivity of SARS-CoV-2 based on untargeted host-removed mNGS technology. 25 samples were selected and assayed using two or more different sequencing depths (M), the relevance between sequencing depths and SARS-CoV-2 detection reads was exhibited in **Figure 5**. The SARS-CoV-2 detection reads of 7 samples (**Figures 5A–G**) showed low a Ct value at around 40 based on RT-PCR were consistently negative (y=0) for mNGS, even when the sequencing depths were improved up to sixfold for Sample-34 (**Figure 5F**). Only one sample witch from negative (y=0) to positive (y=1) (**Figure 5H**). Further investigations on 17 samples with positive results for mNGS detection showed that there was only slight improvement in SARS-CoV-2 detection read even when the sequencing depths were enhanced for several folds (**Figures 5I–Y**). To some extent, these results demonstrate that the enhancement of sequencing depths will not yield much improvement for SARS-CoV-2 detection reads.

### Determining the compositions of both the fungal and bacterial communities by untargeted host-removed mNGS

**Figure 6:**
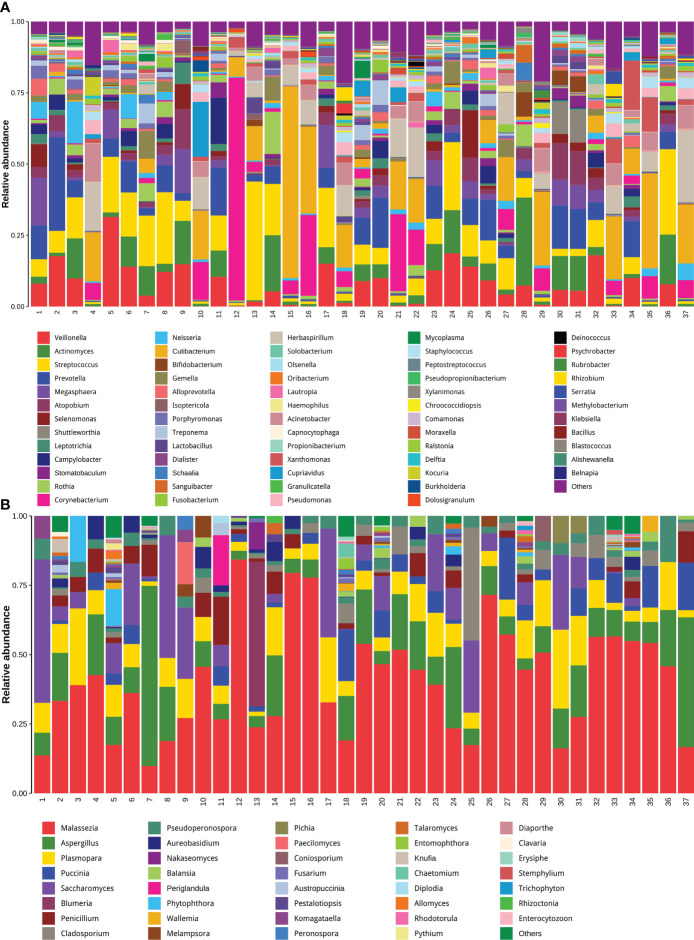
Untargeted host-removed mNGS accurately revealed the compositions of both the bacterial and fungal and bacterial communities. The bacterial **(A)** and fungal **(B)** with abundance greater than 1% were presented, and less than 1% of the total abundance were combined into the “Other <1%” category.

Another important aspect of data analysis of such metagenomic data from untargeted host-removed mNGS is determining the microbial composition and quantifying the microbial abundances based on the metagenomic sequencing data. Until now, most studies have determined respiratory microbial composition using 16S ribosomal DNA (16S rDNA) gene sequencing, whether the host-removed mNGS possess the same utility has not yet been fully explored. The entire sequence alignment from all 37 samples is presented in **Figures 6A, B**, we found that the bacterial community structures determined by mNGS were highly abundant with various bacterial, fungal and bacterial species. More importantly, mNGS can comprehensively reveal the normal upper respiratory tract flora, such as Veillonella, Actinomyces, Streptococcus and Prevotella, all recognized as oral commensals (**Figure 6A**) (17, 18). Malassezia, a dominant fungal genus on the human skin and upper airways of most healthy people and related to human autoimmunity and skin diseases (19–21), was detected in all samples. Aspergillus species, one of the most common pathogenic fungi causing upper and lower airway disorders, was also detected in all samples (**Figure 6B**) (22). These results demonstrated that the bacterial and fungal community structures revealed by mNGS are similar to the microbial communities commonly reported in previous studies based on other technologies like 16S rDNA gene sequencing.

### Functional enrichment analysis of DEGs and xCell score between patients with high and low virus load

**Figure 7:**
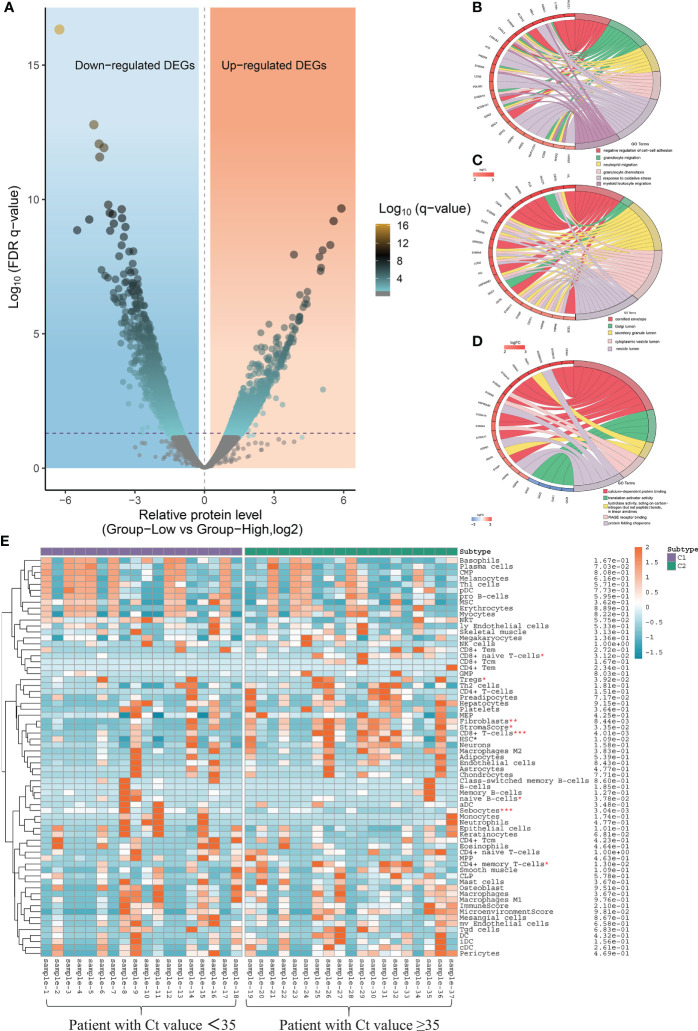
Local nasopharyngeal immune microenvironment analysis between high and low virus load patients. **(A)** Differentially expressed genes (DEG) by volcano diagram. **(B)** Functional enrichment analysis of the biological process (BP) **(C)** Functional enrichment analysis of cellular component (CC). **(D)** Functional enrichment analysis of molecular function (MF). **(E)** xCell immune score identifies the difference in abundance scores of 64 immune cell types between patients with high and low viral load.

Taking the CT value of 35 as the critical point, a total of 462 differentially expressed genes (DEGs) were identified between patients with high and low CT values of SARS-CoV-2, including 127 up-regulated and 335 down-regulated genes (**Figure 7A**). To determine the functional annotation of the DEGs between patients with high and low CT values of SARS-CoV-2, we performed the Gene Ontology (GO) analysis and presented the expression levels of genes in each term in a GO circle plot using the R package GO plot. Based on GO enrichment analysis, DEGs were divided into the three principal GO organization categories: biological process (31 genes) (**Figure 7B**), cellular component (25 genes) (**Figure 7C**), and molecular function (31 genes) (**Figure 7D**). Next, we analyzed the xCell score using the R package ‘xCell’ (https://github.com/dviraran/xCell). xCell is a newly published method based on ssGSEA that estimates the abundance scores of 64 immune cell types, including adaptive and innate immune cells, epithelial cells, hematopoietic progenitors, and extracellular matrix cells. Based on the comparison between 18 patients with high viral load and 19 patients with low viral load, we found that the cellular proportions of CD4^+^ memory T cells, CD8^+^ naive T cells, CD8^+^ T cells, Fibroblasts, HSC, Microenvironment Score and Stroma Score in patients with low viral load were significantly higher than that in patients with high viral load; while the Sebocytes have a higher proportion in patients with high viral load (*p*<0.05) (**Figure 7E**) (**Supplementary Figure S1**).

## Discussion

mNGS is a revolutionary diagnostic tool capable of simultaneously detecting all microbial and host gene expressions. However, it is not very clear what the sensitivity level of this technology is compared with traditional detection methods, especially PCR. In the present study, we systematically evaluated the sensitivity of untargeted host-removed mNGS between the mNGS and two approved qRT-PCR methods. Further series analyses revealed that mNGS have superior performance in the comprehensive identification of the pathogen and simultaneously reveal the transcriptional profiles of the microbiome and host responses.

In recent years, mNGS has emerged with more rapid and accurate diagnostic advantages than traditional methods, especially in culture-negative samples. The sensitivity of mNGS varies in different kinds of pathogens, and while many studies have demonstrated that mNGS is more sensitive than conventional culture (9, 23, 24), there has not been fully proven that whether mNGS could yield high sensitivity than qPCR, which is widely considered as the high-sensitivity. To stringently evaluate the sensitivity of mNGS, 37 samples confirmed to be positive by two different RT-PCR kits simultaneously were used to assayed by mNGS. Results showed that the detection rate of mNGS achieved 92.9% (26/28) for 28 samples with Ct value ≤ 35 and 81.1% (30/37) for all 37 samples (**Figure 1**), this compliance was similar to other studies (25). A recent study demonstrated that the infectious virus will no longer be isolated from the patients with Ct value of qPCR>35 and there was no viral shedding from infectious patients when the Ct value was>28 (26). In view of this, the detection rate of mNGS would meet clinical need and diagnosis of COVID-19.

Recent advances and the growing popularity of mNGS have enabled the rapid identification and traceability of emerging infectious diseases in basic medical institutions or medical laboratories worldwide. The three major pathogenic human coronaviruses (CoVs) are the SARS-CoV, the Middle East respiratory syndrome (MERS)-CoV and SARS-CoV-2 (27). The nucleotide sequence of SARS-CoV-2 is ∼79% similar to SARS-CoV-1 and about 50% with MERS-CoV (Middle East respiratory syndrome coronavirus) (28). In addition, SARS-CoV-2 is genetically very similar to other Coronaviruses (29), so fully understanding its RNA sequence is the key to identifying SARS-CoV-2, especially in the early stage of the epidemic. Our research demonstrated that the mNGS can could not only accurately diagnose COVID-19, but also simultaneously achieve traceability at the first time point.

The sensitivity of mNGS for detecting pathogen-derived genomes could be improved by increasing sequencing depth or decreasing the high human host DNA background (8). However, the effect of the removal of host cell-derived nucleic acids is still controversial, especially for samples with low microbial content, the removal of human host DNA background can significantly reduce the detection rate of target microorganisms (30). Different strategies of host removal may cause different outcomes for various pathogens; further research with larger sample sizes is needed to define its clinical utility. Our data provided the first evidence that removing human host DNA can improve the detection sensitivity for SARS-CoV-2 in swab samples (**Figure 3A**). More interestingly, our study also first demonstrates that the removal of the host does not affect the species abundance of microbes reflected by mNGS (**Figure 4**), indicating that this method can be reliably used to study the microbiome community structure and function.

Hospitalized COVID-19 patients often present with a large spectrum of clinical pictures—from only mild upper respiratory symptoms to severe disease characterized by pneumonia, acute respiratory distress syndrome, and even diverse systemic effects impacting various tissues (31). How infection influences spread from the upper respiratory tract to the lower respiratory tract and cause respiratory failure remains incompletely understood. Recently, an increasing number of researchers are starting to focus on studying the host local immune characteristics related to the SARS-CoV-2 infection based on detecting and analyzing the nasopharyngeal samples from COVID-19 patients (32, 33). It has been demonstrated that SARS-CoV-2 infection can induce unique host immune responses different from infection caused by other respiratory viruses (34, 35). Similar result was also confirmed with the data in our study, which found that there are 462 DEGs between patients with high and low CT values of SARS-CoV-2 (**Figure 7A**).

Further GO enrichment analyses showed that the BP clusters were primarily enriched in functions related to the induction of the inflammasome pathway including granulocyte migration, neutrophil migration, granulocyte chemotaxis and myeloid leukocyte migration (**Figure 7B**). In view of the above series of analysis results, we speculated that differentiating protective host mechanisms might support rapid viral clearance or spread from limited local nasopharynx infection to severe and fatal outcomes. In addition, local nasopharyngeal immune microenvironment analysis also indicated that patients with low viral load presented more intense immune responses than patients with high viral load (**Figure 7E**). This result may also suggest that the host transcriptomic profiling of the host, utilized alone or in combination with the detection results of SARS-CoV-2 from qRT-PCR, is characterized by the ability to establish close connections between viral load and host immune response in the nasopharynx. However, the limitation of this study was the small number of subjects. Future research with larger sample sizes is needed to investigate the relationship between viral load and host immune characteristics and identify biomarkers from the point of view of the human host for distinguishing the degree of infection progress.

In summary, our study offered the first comprehensive description of the practical application and value of untargeted host-removed mNGS for SARS-CoV-2 identification, as well as a comprehensive analysis of the genomic epidemiology of SARS-CoV-2 and the transcriptional profiles of the host responses and microbiome, simultaneously.

## Data availability statement

Metagenome sequencing data data for this study is deposited in the NCBI SRA BioProject repository with the accession PRJNA894695. All other relevant data generated in this manuscript that support the findings of this study are available upon request from the authors. Source data are provided with this paper.

## Ethics statement

The studies involving human participants were reviewed and approved by Shijiazhuang People’s Hospital Ethics Committee. The Ethics Approval Number: [2020]-046.

## Author contributions

SZ, SG and PJ contributed equally to this work. SZ, SG, PJ and LL collected the samples. SZ, MX, CX performed the qPCR and mNGS experiments, SZ and ZY analyzed the data. SZ and WM wrote manuscript. All authors contributed to the article and approved the submitted version.

## Funding

This work was supported by Science and Technology Research and Developmental Guidance Program of Shijiazhuang City (No. 201460503A).

## Conflict of interest

The authors declare that the research was conducted in the absence of any commercial or financial relationships that could be construed as a potential conflict of interest.

## Publisher’s note

All claims expressed in this article are solely those of the authors and do not necessarily represent those of their affiliated organizations, or those of the publisher, the editors and the reviewers. Any product that may be evaluated in this article, or claim that may be made by its manufacturer, is not guaranteed or endorsed by the publisher.
