# Supplementary material for: Clinical characteristics of the host DNA-removed metagenomic next-generation sequencing technology for detecting SARS-CoV-2, revealing host local immune signaling and assisting genomic epidemiology
Source: Front Immunol. 2022 Nov 15;13:1016440. doi: 10.3389/fimmu.2022.1016440 (PMC9705594; doi:10.3389/fimmu.2022.1016440)
Supplement: Supplementary file 1 [file Table_1.pdf]

The detail results of 46 swab samples derived from mNGS and two RT-qPCR detections are given in Table, below. The sequence from Sample-1 to Sample-47

Samples ID	mNGS results		RT-PCR results			
	Detection reads of 2019-nCoV	Total reads	Ct Values of N gene		Ct Values of ORF1ab gene	
			DAAN	BioGerm	DAAN	BioGerm
Samples-1	5653	122	20.89	20.48	20.84	23.09
Samples-2	243	118	25.08	23.12	26.34	27.37
Samples-3	49	87	29.19	31.54	28.37	32.44
Samples-4	13	133	29.77	28.46	30.35	29.99
Samples-5	20	133	32.56	29.14	33.66	33.42
Samples-6	36	406	31.58	29.35	33.37	34.49
Samples-7	103	176	30.83	29.48	31.47	32.16
Samples-8	246	119	29.55	29.80	31.25	31.34
Samples-9	11	440	32.12	30.22	33.26	33.14
Samples-10	2	100	32.11	30.68	33.16	33.66
Samples-11	18	171	32.65	31.05	33.15	34.14
Samples-12	21	141	33.23	31.11	34.16	34.73
Samples-13	50	106	32.36	31.26	33.16	32.94
Samples-14	4	173	33.29	31.81	34.48	36.35
Samples-15	0	113	36.40	31.97	Negative	35.40
Samples-16	159	138	33.48	32.11	35.88	34.00
Samples-17	19	79	33.00	32.16	38.94	34.91
Samples-18	908	117	34.83	32.33	35.26	33.07
Samples-19	0	130	33.25	32.42	34.63	36.08
Samples-20	20	85	35.31	32.51	36.44	34.00
Samples-21	11	47	36.86	33.02	36.15	35.97
Samples-22	5	100	35.00	33.05	33.06	35.19
Samples-23	13	92	35.50	33.12	37.38	Negative
Samples-24	2	88	34.87	33.27	35.12	35.58
Samples-25	1	89	34.91	33.42	37.15	38.05
Samples-26	3	120	36.03	33.52	33.45	Negative
Samples-27	7	95	35.85	34.64	38.02	37.11
Samples-28	19	66	35.00	34.67	36.00	35.56
Samples-29	3	95	37.02	35.14	Negative	40.16
Samples-30	0	60	37.26	35.63	37.49	Negative
Samples-31	0	120	36.58	35.67	37.63	37.22
Samples-32	9	177	33.09	35.73	35.56	Negative
Samples-33	0	112	39.17	36.23	Negative	Negative

Samples-34	0	110	Negative	36.59	37.28	41.33
Samples-35	0	150	36.41	37.60	Negative	37.85
Samples-36	8	90	39.03	Negative	Negative	Negative
Samples-37	5	83	38.95	Negative	Negative	Negative
Samples-38	0	82	Negative	Negative	Negative	Negative
Samples-39	0	180	Negative	Negative	40.18	Negative
Samples-40	0	150	Negative	Negative	Negative	Negative
Samples-41	0	197	Negative	Negative	Negative	Negative
Samples-42	0	119	Negative	Negative	Negative	Negative
Samples-43	0	71	Negative	Negative	Negative	Negative
Samples-44	0	178	Negative	Negative	Negative	Negative
Samples-45	0	94	Negative	Negative	Negative	Negative
Samples-46	0	239	Negative	Negative	Negative	Negative
