# Supplementary figures and images for: Clinical characteristics of the host DNA-removed metagenomic next-generation sequencing technology for detecting SARS-CoV-2, revealing host local immune signaling and assisting genomic epidemiology
Source: Front Immunol. 2022 Nov 15;13:1016440. doi: 10.3389/fimmu.2022.1016440 (PMC9705594; doi:10.3389/fimmu.2022.1016440)

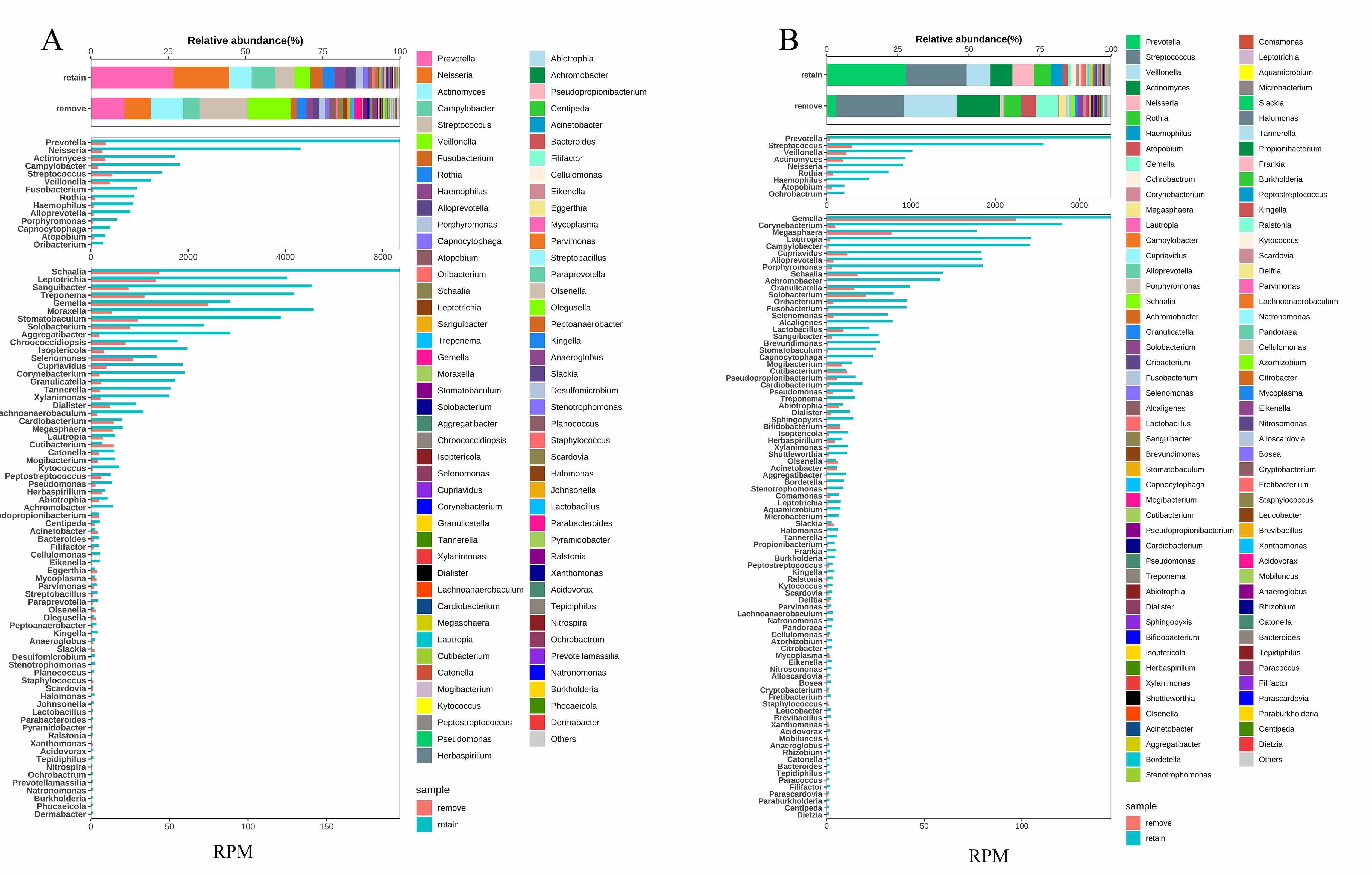

Supplement: Supplementary file 2 [file Image_1.jpeg]

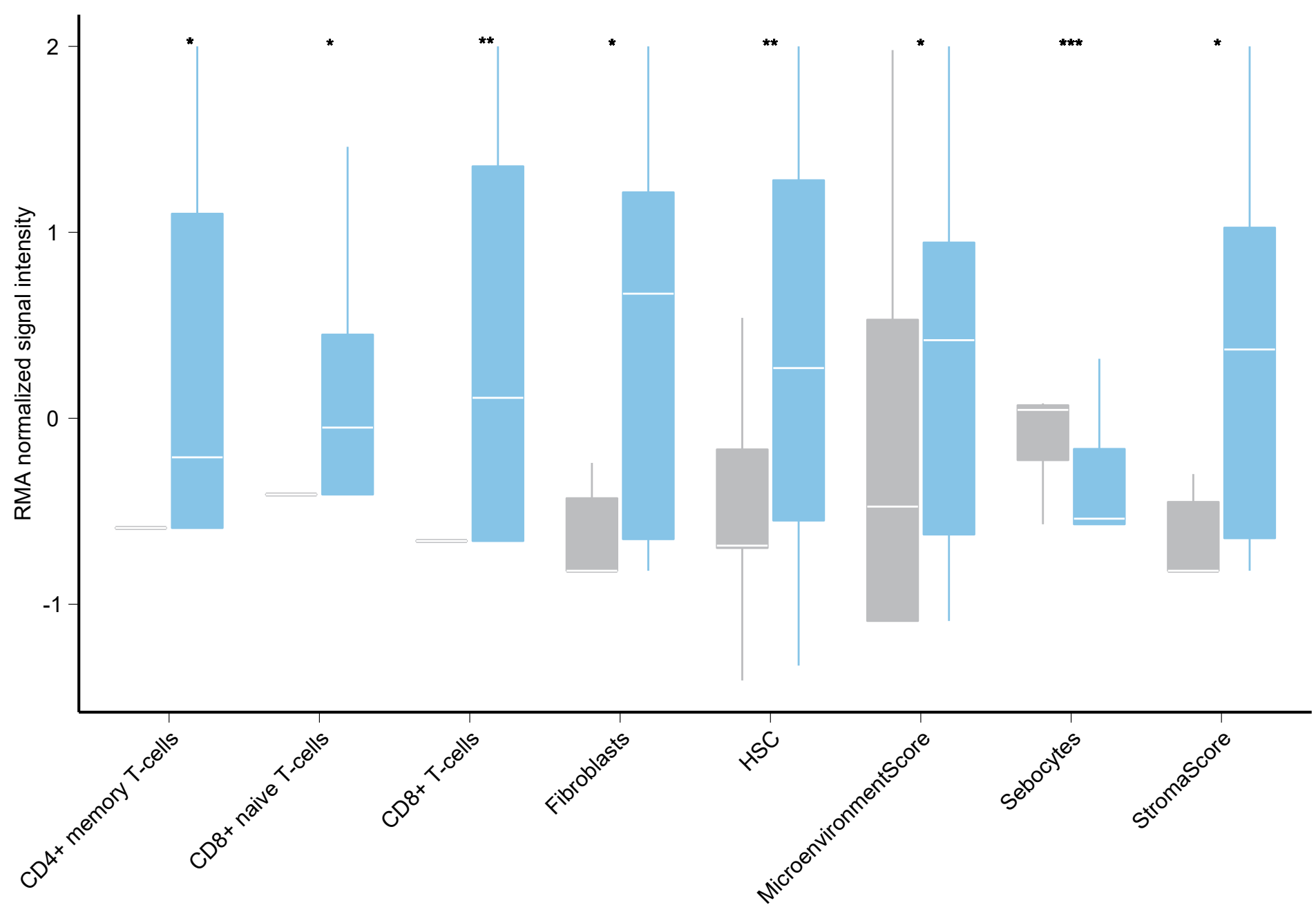

Supplement: Supplementary file 3 [file DataSheet_1.pdf]
